# The influence of antibiotics on transitory resistome during gut colonization with CTX-M-15 and OXA-162 producing *Klebsiella pneumoniae* ST15

**DOI:** 10.1038/s41598-021-85766-6

**Published:** 2021-03-18

**Authors:** Balázs Stercz, Ferenc B. Farkas, Ákos Tóth, Márió Gajdács, Judit Domokos, Viola Horváth, Eszter Ostorházi, Nóra Makra, Béla Kocsis, János Juhász, Balázs Ligeti, Sándor Pongor, Dóra Szabó

**Affiliations:** 1 Institute of Medical Microbiology, Semmelweis University, Nagyvárad tér 4., 1089 Budapest, Hungary; 2 Department of Bacteriology, Mycology and Parasitology, National Public Health Centre, Albert Flórián út 2-6., 1097 Budapest, Hungary; 3 Department of Pharmacodynamics and Biopharmacy, Faculty of Pharmacy, University of Szeged, Eötvös utca 6., 6720 Szeged, Hungary; 4 Department of Inorganic and Analytical Chemistry, Budapest University of Technology and Economics, Szent Gellért tér 4., 1111 Budapest, Hungary; 5 MTA-BME Computation Driven Chemistry Research Group, Szent Gellért tér 4., 1111 Budapest, Hungary; 6 Faculty of Information Technology and Bionics, Péter Pázmány Catholic University, Práter utca 50/A., 1083 Budapest, Hungary

**Keywords:** Microbiology, Molecular biology, Medical research

## Abstract

Great efforts have been made to limit the transmission of carbapenemase-producing *Enterobacteriaceae* (CPE), however, the intestinal reservoir of these strains and its modulation by various antibiotics remain largely unexplored. Our aim was to assess the effects of antibiotic administration (ampicillin, ceftazidime, ciprofloxacin) on the establishment and elimination of intestinal colonization with a CTX-M-15 ESBL and OXA-162 carbapenemase producing *Klebsiella pneumoniae* ST15 (KP5825) in a murine (C57BL/6 male mice) model. Whole genome sequencing of KP5825 strain was performed on an Illumina MiSeq platform. Conjugation assays were carried out by broth mating method. In colonization experiments, 5 × 10^6^ CFU of KP5825 was administered to the animals by orogastric gavage, and antibiotics were administered in their drinking water for two weeks and were changed every day. The gut colonization rates with KP5825 were assessed by cultivation and qPCR. In each of the stool samples, the gene copy number of *bla*_OXA-162_ and *bla*_CTX-M-15_ were determined by qPCR. Antibiotic concentrations in the stool were determined by high pressure liquid chromatography and a bioanalytical method. The KP5825 contained four different plasmid replicon types, namely IncFII(K), IncL, IncFIB and ColpVC. IncL (containing the *bla*_OXA-162_ resistance gene within a Tn1991.2 genetic element) and IncFII(K) (containing the *bla*_CTX-M-15_ resistance gene) plasmids were successfully conjugated. During ampicillin and ceftazidime treatments, colonization rate of KP5825 increased, while, ciprofloxacin treatments in both concentrations (0.1 g/L and 0.5 g/L) led to significantly decreased colonization rates. The gene copy number *bla*_OXA-162_ correlated with *K. pneumoniae *in vivo*,* while a major elevation was observed in the copy number of *bla*_CTX-M-15_ from the first day to the fifteenth day in the 0.5 g/L dose ceftazidime treatment group. Our results demonstrate that commonly used antibiotics may have diverse impacts on the colonization rates of intestinally-carried CPE, in addition to affecting the gene copy number of their resistance genes, thus facilitating their stable persistance and dissemination.

## Introduction

Multidrug-resistant (MDR) Gram-negative bacteria have emerged as a major public health threat. Extended-spectrum β-lactamase (ESBL)-producing *Enterobacteriaceae* have disseminated worldwide and have become a serious concern for clinicians, due to limited therapeutic options, both in community-acquired and nosocomial infections^1^. In the last decade, CTX-M-type ESBLs have replaced TEM- and SHV-types among clinical *Enterobacteriaceae* isolates^2^. The explosive dissemination of CTX-M-type β-lactamases around the world has been referred to as the “CTX-M pandemic”, associated with their increasing description around the globe^3^, and their prevalence rates may vary among different members of the *Enterobacteriaceae* family; nevertheless, they are most common in species, such as *Klebsiella pneumoniae* and *Escherichia coli*^4^. The increasing prevalence of infections caused by MDR Gram-negative bacteria (especially ESBL-producers) was accompanied with the rise in the use of carbapenems for the treatment of these infections^5^. Subsequently, this has further enhanced the emergence and dissemination of carbapenemase-producing *Enterobacteriaceae* (CPE). Although resistance rates to carbapenems remain low in some parts of Europe, the developments in southern and southeastern Europe (which were previously characterized by an unrestricted use of these life-saving drugs) is concerning^6^.

CPE infections are associated with high morbidity and mortality, particularly in vulnerable patient populations, including infants, children and the elderly, hospitalized patients, immuncommpromised patients, as well as the critically ill. The major driving force in the uncontrolled dissemination of these strains is their ability to survive and spread rapidly in healthcare environments; in fact, carbapenemase-production is usually linked to successful MDR clones, commonly associated with nosocomial infections^7,8^. The carbapenemase genes in *Enterobacteriaceae* have been shown to be associated with mobile genetic elements including plasmids or transposons, allowing for the transfer among different members of the family. OXA-48-like carbapenemases are one of the most common carbapenemases (with increasing prevalence in Europe, although wide-ranging differences in their geographic distribution may be observed) in *Enterobacteriaceae*, and they are continuously being introduced into regions of non-endemicity, where they may be responsible for nosocomial outbreaks^6,9^. While *K. pneumoniae* is the main reservoir of *bla*_OXA-48_, the number of studies reporting cases due to other *bla*_OXA-48_ producing *Enterobacteriaceae* species is increasing worldwide^8,10,11^.

Due to the high prevalence and pervasiveness of *bla*_OXA-48_-like carbapenemases in community-associated and nosocomial Gram-negative bacteria, limiting the additional spread of pathogens producing these enzymes is a difficult task^11,12^. *Bla*_OXA-48-like_/*bla*_OXA-48_ carbapenemases are found on plasmids that have a high propensity to disseminate among various bacterial species via horizontal gene transfer (HGT)^9^. It is not uncommon to detect different bacteria containing identical plasmids harboring *bla*_OXA-48_, obtained from the same patient, both as colonizers or as causative agents of infections^13^. OXA-48 is associated with different *Tn1999* transposon variants and located mainly as the only antibiotic resistance gene on the conjugative IncL (IncL/M) replicon type plasmids^14,15^. The occurrence of pOXA-48a-like IncL plasmids were described in many Gram-negative bacteria, including *Citrobacter freundii, E. coli*, *Enterobacter cloacae*, *K. pneumoniae*, *K. oxytoca* and *Raoultella planticola*^16,17^*.* Some high-risk clones (e.g., ST11, ST15, ST101 and ST307 for *K. pneumoniae,* and ST38 and ST410 for *E. coli*) have been associated with the global dispersal of many OXA-type carbapenemases (OXA-48, OXA-181, OXA-232 and OXA-204)^13,18–20^. OXA-162—which is also a member of the OXA-48-like carbapenemases—has been observed in different gut bacteria, reported from Turkey, Germany, Greece and Hungary until now^10,21–23^.

**Figure 1:**
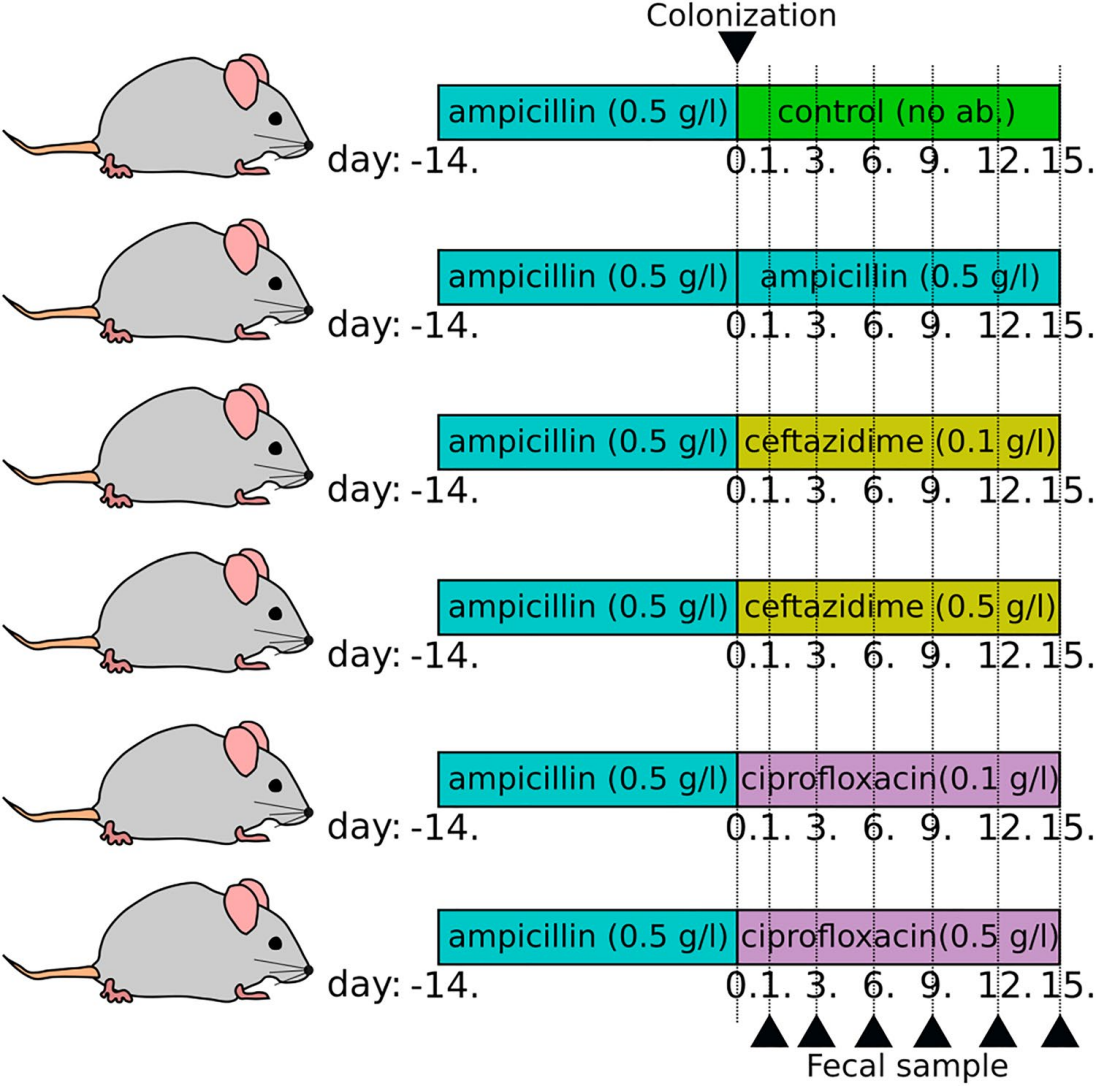
The colonization protocol used in our experiments.

*Enterobacteriaceae* are inhabitants of human gut microbiota, and feacal carriers may represent an important reservoir for person-to-person transmission and dissemination of bacteria. Furthermore, gut colonization by MDR bacteria has been associated with a high risk of developing subsequent clinical infection associated with increased mortality^7,24^. Therefore, active surveillance is a key part in preventing the spread of such strains. Efforts to limit the transmission of carbapenemase-producing *K. pneumoniae* strains focus on basic and enhanced infection control measures, while the importance of the intestinal reservoir of these strains and its modulation by various antibiotics remain largely unexplored^25^. Administration of antibiotics is a known risk factor for the development of resistance, however its role in colonization is still unclear. In this study, our aim was to assess the effects of antibiotic administration on the establishment and elimination of intestinal colonization with a CTX-M-15 ESBL and OXA-162 carbapenemase co-producing *K. pneumoniae* in a murine model, followed by administration of ampicillin, ceftazidime or ciprofloxacin (Fig. 1).

## Results

Based on the whole genome sequencing the KP5825 strain harboured *bla*_CTX-M-15_ ESBL and the *bla*_OXA-162_ carbapenemase, as well as other antibiotic resistance-determinants for β-lactam resistance (*bla*_SHV-28_ and *bla*_OXA-__1_). The KP5825 isolate harboured several chromosomal nucleotid mutations resulted in GyrA amino acid alterations in position Ser83Phe, Asp87Ala and Asn645His and in ParC in position Ser80Ile and Pro402Ala, furthermore the isolate also carried the plasmid-borne *aac(6′)-Ib-cr* fluoroquinolone resistance determinant. The isolate carried resistance-determinants for aminoglycoside resistance (*aac(3)-IIa, aph(3′)-Ia* and *aac(6′)Ib-cr* as well*.* In addition, four different plasmid replicon types, namely IncFII(K), IncL, IncFIB and ColpVC were detected in the KP5825. The IncL and IncFII plasmids were successfully conjugated, and the IncL plasmid contained only the *bla*_OXA-162_ resistance gene within a *Tn1991.2* genetic element, while the IncF(II)K contained the *bla*_CTX-M-15_ resistance gene.

The KP5825 showed high level resistance against the beta-lactam and fluoroquinolone antibiotics based on MIC values determined in the broth microdilution assay. The broth mating procedure-based in vitro conjugation assay performed was successful, and the conjugated *E. coli* J53 harbouring the pOXA-162 showed increased resistance in case of ertapenem and meropenem and in case of *E. coli* J53 harbouring pCTX-M-15, resistance to cephalosporins (ceftazidime, cefotaxime) was detected. The characteristics of KP5825 and the conjugated *E. coli* J53 strains are shown in Table 1.

**Table 1:** Features of KP5825 and the transconjugated *E. coli* J53 strains.

	Strains			
	KP5825	*E. coli* J53	*E. coli* J53 Transconjugant-5825/1 pCTX-M-15	*E. coli* J53 Transconjugant-5825/2 pOXA-162
Plasmid replicon types	IncF(I)B, IncF(II)K, ColpVC, IncL	NA	IncF(II)K	IncL
**Beta-lactamases**		NA	*bla*CTX-M-15	*bla*OXA-162
Chromosomal	*bla*SHV-28			
On mobile genetic elements	*bla*OXA-1, *bla*OXA-162, *bla*CTX-M-15			
Mobile genetic elements	*Class I, Tn1999.2,*	NA	*Class I*	*Tn1999.2*
**Quinolone resistance determinants**		NA	*aac(6′)Ib-cr*	
Chromosomal	*gyrA (S83F, D87A, N645H)*			
	*parC (S80I, P402A)*			
On mobile genetic elements	*aac(6′)Ib-cr*			
Other resistance genes	*aac(3)-IIa, aph(3′)Ia*	NA		
**MIC (mg/L)**				
Ampicillin	> 32	4	> 32	> 32
Ceftazidime	> 32	0.25	> 32	0.25
Cefotaxime	> 32	0.125	> 32	0.25
Ertapenem	> 32	< 0.0625	< 0.0625	0.25
Imipenem	16	0.5	0.5	0.5
Meropenem	> 32	< 0.0625	< 0.0625	0.125
Ciprofloxacin	> 32	< 0.0625	< 0.0625	< 0.0625

MIC: minimum inhibitory concentrations; NA: not applicable.

In the colonization studies with 6–8 week-old C57BL/6 male mice the antibiotics were administered in their drinking water for two weeks. The concentration of antibiotics in mouse stool was assessed with high pressure liquid chromatography (HPLC) on the 1st and 15th day after KP5825 colonization. The ampicillin concentration in the stool samples in Amp_0.5 group on the first day was 720.2 ± 247.0 µg/g (average ± SD), while on the fifteenth day 739.3 ± 219.4 µg/g. The average ciprofloxacin concentration in the Cip_0.1 group was 17.2 ± 5.96 µg/g on the first day, and 20.7 ± 4.97 µg/g on the fifteenth day; and in Cip_0.5 group, it was 203.8 ± 46.0 µg/g on the first day and 244.8 ± 61.9 µg/g on fifteenth day. Ceftazidime was undetecable from mice stool samples.

**Figure 2:**
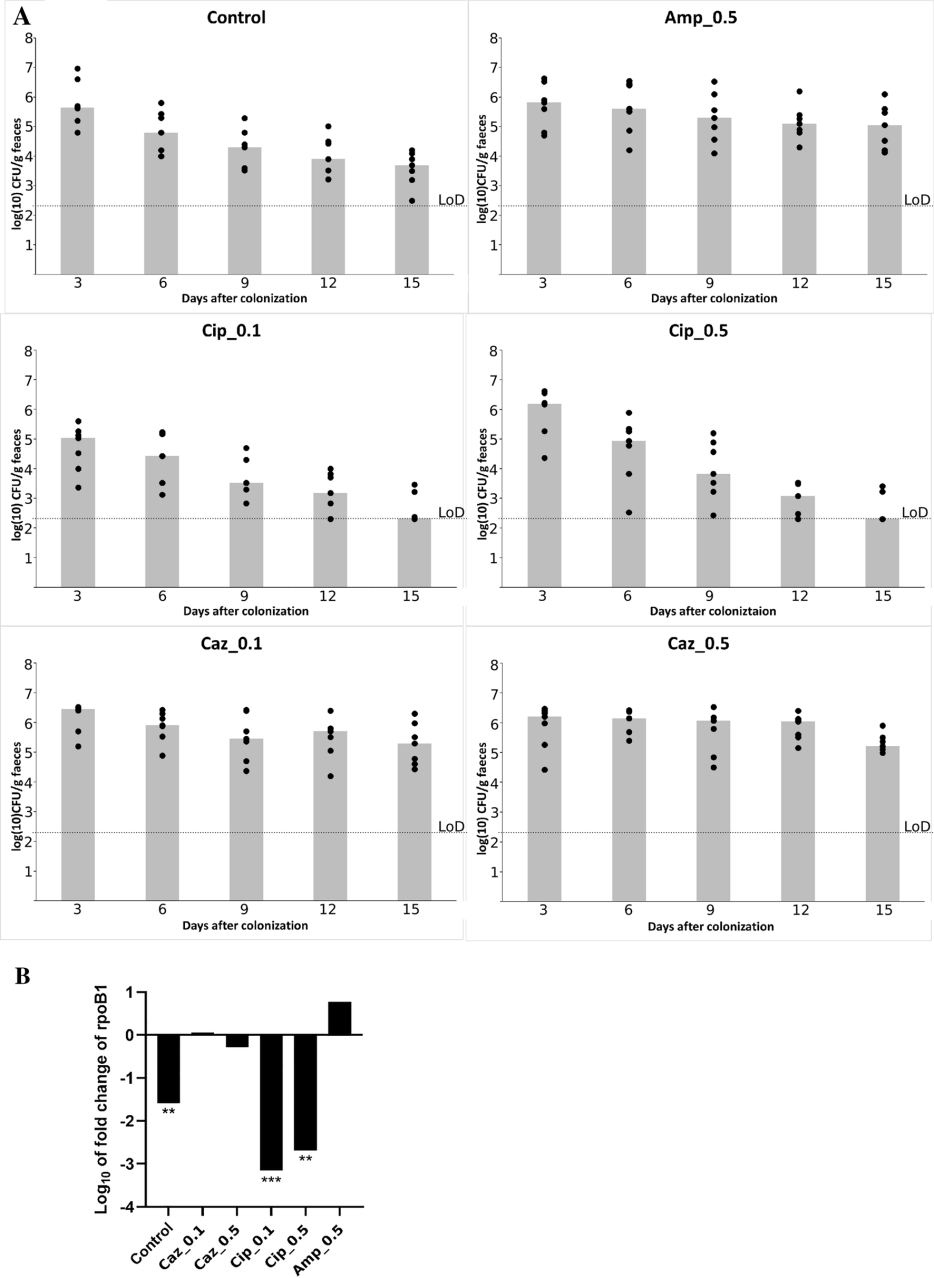
(**A**) Effect of various antibiotic administration on the establishment of intestinal colonization with KP5825 by orogastric gavage on day 0 (n = 7 mice per antibiotic treatment group). Densities of KP5825 are shown on 3rd, 6th, 9th, 12th and 15th days after colonization. The limit of detection (LoD) (~ 2.3 log_10_ CFU/g) was assigned. Colums represent median values. (**B**) Changes in the relative colonization rate by KP5825 in the antibiotic-treated groups and in the control group. The log_10_ fold change of *rpoB1* housekeeping gene show the relative colonization rate between the first and fifteenth day of the colonization with the different treatment.

The colonization was performed with the KP5825 strain administered by orogastric gavage and the colonization rate of mice with CTX-M-15 ESBL- and OXA-162 carbapenemase-producing KP5825 was quantified by both a conventional culture analysis method and the qPCR technique, in order to simultaneously determine the absolute and relative colonization rates with the tested isolate. The effect of different treatment regimens on colonization with KP5825 are shown in Fig. 1A,B. The densities of KP5825 detected in feces were assayed on the 3rd, 6th, 9th, 12th and 15th days. If KP5825 organisms were not detected in the stool, the lower limit of detection (~ 2.3 log_10_ CFU/g) was assigned. In case of all observation periods of ceftazidime treatments, the rate of KP5825 colonies were the highest, while on the other hand, during ciprofloxacin treatments they were the lowest (Fig. 2A). During ampicillin (Amp_0.5) and ceftazidime treatments (Caz_0.1 and Caz_0.5), the absolute colonization rate of the carbapenem-resistant KP5825 slightly increased. Upon treatment with ampicillin, a moderate increase of *K. pneumoniae* cell count was detected. In contrast, during ciprofloxacin treatments in both concentrations (Cip_0.1 and Cip_0.5) and in the control group, the colonization rates have decreased significantly. The most extensive decrease in colonization rate was observed in the group treated with the lower dose of ciprofloxacin (Cip_0.1). These alterations are the most unexpected as the present carbapenemase-producing *K. pneumoniae* shows high level resistance to fluoroquinolones. These results were consequent with qPCR results by observing the log_10_ fold change of *rpoB1* housekeeping gene designed for KP5825. The relative colonization rate of KP5825 between the first and fifteenth day of colonization showed also differences between antibiotic treatments. In Caz_0.1 group the colonization rate of carbapenem-resistant KP5825 slightly increased, while on the other hand treatment with ampicillin resulted in a moderate increase of KP5825. An extensive decrease in colonization rate was observed in the groups treated ciprofloxacin (Cip_0.1, Cip_0.5) (Fig. 2B).

**Figure 3:**
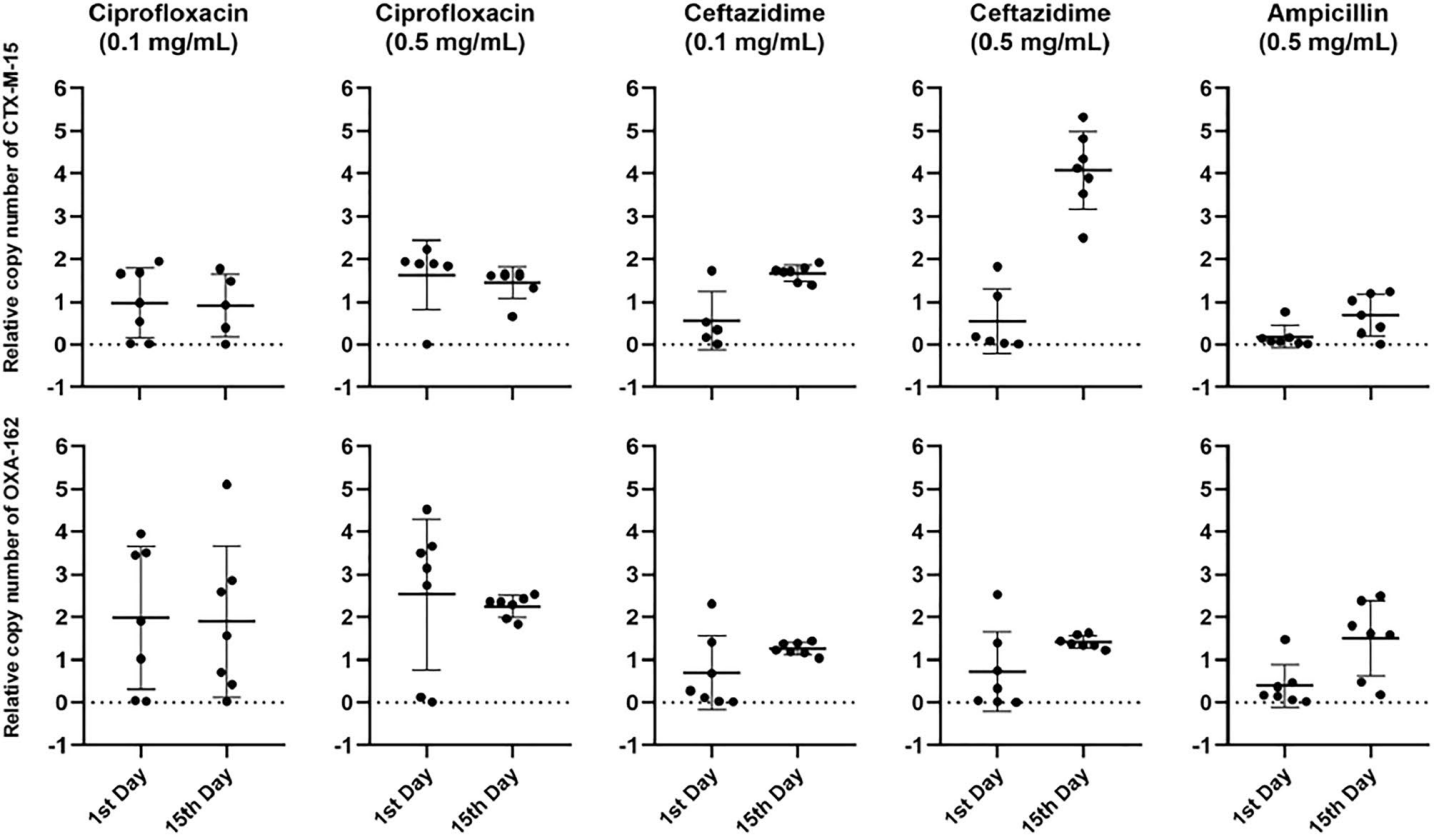
The relative copy number of the *bla*_CTX-M-15_ and *bla*_OXA-162_ genes from the feces of individual mice after the different antibiotic-treatment regiments on the first and fifteenth days after colonization with KP5825.

The effect of antibiotic-treatment regiments on *bla*_CTX-M-15_ and *bla*_OXA-162_ genes’ copy number in the gut was determined by qPCR from each the stool sample and results were calculated as the fold change of gene normalized to the *rpoB1* reference gene and relative to the control mice. The relative copy number of the ESBL *bla*_CTX-M_-_15_ and the carbapenemase *bla*_OXA-162_ were determined and these results were correlated to the *rpoB1* housekeeping gene of KP5825 on the first and on the fifteenth days from the feces of each mouse used in the experiment (Fig. 3). The relative copy number did not change for *bla*_OXA-162_ during the observed period in any treatment group. In contrast, a major elevation was observed from the first day to the fifteenth day in the treatment group with the Caz_0.5 treatment yielding 2 and 400-fold absolute gene copy number increase of the *bla*_CTX-M-15_ gene. At the same time, the relative copy number of the *bla*_CTX-M-15_ gene (which was controlled with the rate of the *rpoB* gene) also increased significantly (p < 0.05) from 2- to 5-times relative to the control in the Caz_0.5 treatment group (Fig. 3). Nevertheless, only the original CTX-M-15 and OXA-162-producing *K. pneumoniae* isolate could be reisolated from various feaces samples from mice during the experiment using the selective CHROMagar plates. We could not isolate other ESBL or carbapenenamse-producing bacteria on the appropriate selective culture media, except the original KP5825.

## Discussion

*Klebsiella pneumoniae* is a prevalent and dangerous cause of hospital-associated infections, especially in ICUs^24,25^. Because of their global spread, high mortality and very limited therapeutic options, carbapenem-resistant *K. pneumoniae* (CRKP) was declared a major public health threat, rated as priority 1, critical pathogen by the World Health Organization^26–28^. Patients with intestinal carriage of CRKP upon admission may act as reservoirs^29^; moreover, gastrointestinal colonization with MDR *K. pneumoniae* increases the risk of subsequent infections and mortality^29,30^. Colonization with a carbapenem-resistant *Klebsiella* has been highlighted as a hallmark of a subsequent extraintestinal infection by these pathogens; therefore, the identification of patients whom are positive for CRKP-colonization may be an important step to introduce infection control interventions and to save patients from developing an infection^31^.

Our experiments aimed to investigate the effects of various antibiotic treatments on the gastrointestinal colonization, gene dynamics and role in the resistome of the high-risk clone *K. pneumoniae* ST15, producing the CTX-M-15 and OXA-162 β-lactamases, focusing on the major problem of the emergence and spread of ESBL and carbepenemase genes. In case of OXA-162, the host plasmid IncL and in case of CTX-M-15, the host plasmid IncFII of the high risk clone *K. pneumoniae* play an important role in its international dissemination. In our experiment, both plasmids were shown to be conjugable. OXA-48-like enzymes itselves hydrolyze carbapenems to a lesser extent, as we also observed, however their co-occurrence with other β-lactam resistance mechanisms, such as membrane impermeability, may result in high-level carbapenem-resistance^9–11^.

Three antibiotics were included in our study, namely ampicillin, ceftazidime (representatives of β-lactams) and ciprofloxacin. Ampicillin and its derivates (i.e. the aminopenicillins) and ciprofloxacin (a member of the fluoroquinolones) are still one the most widely used drugs in the community, therefore, the assessment of their effect on the gut resistome is of utmost importance^32^. Ceftazidime has been recently sidelined in therapy, due to its availability and the emergence of ESBLs worldwide. Nevertheless, the introduction of ceftazidime and avibactam, a novel cephalosporin/β-lactamase inhibitor combination into the clinical practice—especially for the treatment of OXA-48-type (Class D) carbapenemase producing MDR Gram-negative organisms has provided renewed relevance to this drug^33,34^. Results of our experiments have shown that the studied antibiotic treatment regiments affected the resistome of mice in different ways.

Previous antibiotic therapy is an independent risk factor for colonization with ESBLproducing *Enterobacteriaceae* as demonstrated several studies^35,36^. During our studies, ampicillin pre-treatment was used (for a duration of 14 days) to maintain and promote the colonization of KP5825 in all treatment groups, which was done to model the natural colonization of the host with the microorganism. The rationale behind this was that—based on literature findings—gastro-intestinal colonization with *K. pneumoniae* is difficult to establish in mice via gavage treatment and that antibiotic (ampicillin) pre-treatment has been noted to play a role in disrupting the microbiota of the desired host to allow for the colonization of *K. pneumoniae*^37,38^.

There are controversial data regarding the effects of beta-lactam treatment on the gastrointestinal colonization with multi-drug reistant organisms. Several authors have noted that exposure to various β-lactam antibiotics allow for the colonization by ESBLs (an ST131 *E. coli* strain was used in the experiments), regardless of negatively affecting (clindamycin) the members of the Bacteroidales order or not (cefuroxime and dicloxacillin)^39^. Conversely, others reported that the treatment with cephalosporins at the ICU did not increase the acquisition rate of carbapenem-resistant *Enterobacteriaceae*^40^. In our study, as a consequence of the treatment with β-lactam antibiotics, both the colonization rate and—independently from this—the gene copy number of *bla*_CTX-M-15_ both increased. Nevertheless, the copy number of *bla*_OXA-162_ correlated with the colonization rate of KP5825. In the case of *bla*_CTX-M-15_ located on IncF(II)K plasmid, a higher gene copy number was detected in mice stool samples after cephalosporin treatment, thus indicating a shift in resistome. The measurement of the replicon’ copy number could have additionally provided valuable information on the underlying reasion for the observed increase, however, this experiment was unfortunately not performed. Given that the CTX-15-producing transconjugant could not be isolated from stool samples, highlights that either recipient Enterobacteriales was not detectable (or culturable) in feces of mice or the copy number of *bla*_CTX-M-15_ resistance genes was increased only in the host cells. Thus, it may also be assumed that the *bla*_CTX-M-15_ gene may have been transferred to non-culturable bacteria. However, this does not change the fact that *bla*_CTX-M-15_ gene was present in higher levels and the plasmid is capable of conjugation in present of susceptible recipient bacterium.

The fact that ciprofloxacin reduced the colonization rate in our experiments is particularly interesting, especially in light of the fact that the colonizing *K. pneumoniae* strain itself had high-level fluoroquinolone resistance as it had both chromosomal and plasmid-mediated quinolone resistance determinants. Regardless, our carbapenem-resistant *K. pneumoniae* isolate disappeared or its load has significantly decreased in the feces of ciprofloxacin treated mice. These findings support earlier studies where ciprofloxacin did not increase the abundance of antibiotic resistance genes-carrying plasmids and failed to promote colonization with MDR Gram-negative bacteria^37,41^. A potential explanation involves the limited antimicrobial effects of ciprofloxacin on the anaerobic intestinal microbiota^42^.

Based on the results of our experiments, it may be assumed that the differences in the colonization effects of the tested antibiotics are mainly rooted in their structre-activity relationships and biological targets, rather then the doses in which they were applied (there were no difference between different doses of the same antibiotic). These results highlights the fact that the that timing of the antimicrobial adiministration relative to CPE exposure is also an important parameter to consider in providing ecological space for the implantation and expansion of the MDR strain.

## Conclusions

In summary, our results have shown that in the presence of β-lactam antibiotics, the amount of the high-risk clone of *K. pneumoniae* showed an increase in the absolute and relative colonization rate, as well as gene copy number of *bla*_CTX-M-15_ on the IncF(II) conjugative plasmid. In contrast, gene copy of *bla*_OXA-162_—which was also conjugative in vitro on IncL plasmid—correlated with *K. pneumoniae* cell count in vivo. Increases in the degree of colonization in the presence of antibiotics has been described by previous studies, however, a clone-independent change in the copy number of *bla*_CTX-M-15_ resistance genes in vivo has not been previously described. In contrast, a parallel decrease in both the clone and the resistance genes was observed after the treatment of fluoroquinolones. This has already been observed by others, but contrasting observations have also been published. Gastrointestinal colonization of MDR bacteria poses a serious clinical problem, both in community-based and nosocomial settings, and in our study we demonstrated a diverse influence of commonly administered antibiotics (ampicillin, ceftazidime, ciprofloxacin) on intestinally carried multidrug-resistant *K. pneumoniae.*

## Methods

### Bacterial strains

*K. pneumoniae* ST15 (KP5825) was obtained from National Public Health Centre (Budapest, Hungary)^23^. Azide-resistant *E. coli* J53 was used in the conjugation assays.

### Antibacterial susceptibility testing

Antibacterial susceptibility testing was performed by the broth microdilution method according to the EUCAST guidelines v.9.0 (www.eucast.org)^43^. Incubation was performed at 35 °C for 16–20 h and minimum inhibitory concentrations (MICs) were determined visually. *E. coli* ATCC 25922 was used as control strain.

### Conjugation assay

Conjugation assays were carried out by broth mating procedure in Lurian-Bertani (LB) broth (Sigma-Aldrich, USA) with the KP5825 isolate as donor and the *E. coli* J53 azide resistant strain as recipient^43^. Overnight cultures of donor and recipient strains grown in LB broth were added to 8 mL fresh LB broth at a donor-recipient ratio of 1:1 (300 μL of cultures each), and incubated for 4 h at 37 °C. The mixed cultures were centrifuged and the supernatant was removed in order to get rid of the antibiotics, to avoid the inhibitory effect against *E. coli* J53. The pellet was re-suspended in fresh culture and plated onto a LB-agar containing 100 μg/mL azide (Sigma-Aldrich) and 0.1 μg/mL of cefotaxime (Sigma-Aldrich) and/or 0.1 μg/mL of ertapenem (Sigma-Aldrich)^44^. Colonies growing on the selective agar plates and again on subculture agar were subjected to confirmatory tests of ESBLs and carbapenemase by CTX-M Multi and Carba 5 immunochromatographic assays (NG Biotech, Guipry, France).

### Mouse model of in vivo colonization with KP5825

All experiments were carried out using 6–8 week-old C57BL/6 male mice weighted 24–26 g (Jackson Laboratory, Bar Harbor, Maine, USA) and housed in sterile cages with irradiated food and acidified water. Each group contained seven mice. For experiments involving antibiotic treatment, 0.5 g/L of ampicillin (Sandoz GmbH) was administered to animals in the drinking water for fourteen days and changed every day. For colonization experiments, 5 × 10^6^ CFU of *K. pneumoniae* KP5825 was administered by orogastric gavage in a 200 μl volume on the fourteenth and fifteenth day of ampicillin pre-treatment. After the oral colonization with KP5825 the following antibiotics—0.5 g/L ampicillin (Amp_0.5), 0.1 g/L ceftazidime (GlaxoSmithKline) (Caz_0.1), 0.5 g/L ceftazidime (Caz_0.5), 0.1 g/L ciprofloxacin (Bayer AG) (Cip_0.1) and 0.5 g/L ciprofloxacin (Cip_0.5)—were further administered to the animals in the drinking water for two weeks and changed every day (Fig. 1).

Mice were single-housed at the time of colonization experiment. Animals were maintained in a specific pathogen-free facility at Institute of Medical Microbiology, Semmelweis University. All mouse handling, cage changes and feacal pellet collection were performed in a biosafety level 2 (BSL-2) facility, with personnel wearing sterile gowns, masks and gloves.

### Sequencing

Genomic DNA from KP5825 was isolated by NucleoSpin Microbial DNA Kit (Macherey Nagel), and plasmid DNA was isolated by NucleoSpin Plasmid DNA Kit (Macherey Nagel) according to the manufacturer’s instructions. The quality and quantity of isolated DNA was assessed by measurements using a Qubit 4.0 fluorometer (Invitrogen, Waltham, USA) and Tapestation 4150 systems (Agilent, Santa Clara, USA). The NGS libraries were prepared using the Nextera DNA Flex Library Prep Kit (Illumina, Eindhoven, The Netherlands) with Nextera DNA CD Indexes^45^. The NGS libraries were sequenced on an Illumina MiSeq instrument using the MiSeq Reagent Kit v2 using paired end 250 bp reads at the Genomics Resource Center at the Biomi Ltd. The fastq files were imported directly from Illumina BaseSpace to the BioNumerics version 7.6 software’s (Applied Maths NV, Belgium) cloud-based calculation engine^45^. De novo sequence assemblies were made with the SPAdes *de novo* genome assembler (version 3.7.1).

### Accession numbers, data deposition

The genomic assembly of the OXA-162 and CTX-M-15 producing *K. pneumoniae* KP5825 have been deposited at European Nucleotide Archive at study PRJEB38863. The assembly of the plasmid containing the OXA-162 submitted under ERZ1461529 accession number and the plasmid containing the CTX-M-15 submitted under ERZ1462751 accession number to the European Nucleotide Archive.

### Determination of the antibiotic concentrations in the fecal samples of mice

The concentrations of antibiotics in the stool samples of each mice were determined by HPLC at two different time points: on the first and fifteenth day after colonization with KP5825. For the determination of ampicillin, mouse fecal pellets were extracted with acetonitrile–water mixture after homogenization and derivatized with formaldehyde. The fluorescent derivative was separated on a Phenomenex Kinetex EVO C18 column and detected at λ_ex_ = 346 nm and λ_em_ = 422 nm wavelenghts. Ciprofloxacin was extracted from mouse faeces with 0.1 M phosphoric acid. The sample extract was separated on the same column and detected at λ_ex_ = 310 nm and λ_em_ = 445 nm wavelenghts using fluorescent detection. Ceftazidime was extracted with water and separated on an Agilent Polaris 3 C18-Ether column followed by UV detection at 261 nm.

### Assessment of the colonization rate with KP5825 by cultivation during different antibiotic treatments

To quantify the burden of KP5825, fresh stool samples were collected on the 3rd, 6th, 9th, 12th and 15th days after the colonization with KP5825. Fresh stool specimens were used for the quantitative culture of KP5825. Serially diluted aliquots were inoculated onto a selective CHROMagar (Mast Diagnostika, Reinfeld, Germany) containing 0.1 μg/mL cefotaxime. Plates were incubated at 37 °C for 48 h and the CFU per gram of stool was calculated. The color and morphological characteristics of the colonies grown were assessed on CHROMagar (Mast Diagnostika) after 24 h and 48 h of incubation in ambient air at 35 °C.

### Assessment of the colonization rate with KP5825 and copy number of bla_CTX-M-15_ and bla_OXA-162_ by qPCR assay during different antibiotic treatments

Genomic DNA of KP5825 was extracted by QiaAmp Power fecal kit (QIAGEN, Venlo, NL) strictly based on manufacturer protocols. Oligonucleotid primers and FAM (fluorescein amidite)- and VIC (2′-chloro-7′phenyl-1,4-dichloro-6-carboxy-fluorescein)-labelled probes were designed by Primer Express 3.0 software (Table 2). The qPCR was carried out in a Step One Real-Time PCR System (Applied BioSystems, Thermo Fisher Scientific) in default setting. The copy number of resistance gene results were evaluated using the 2^−ΔΔCt^ method^46^. Utilizing the 2^−ΔΔCt^ method, results are presented as the fold change of gene normalized to the *rpoB1* reference gene and relative to the control mice. The number of *rpoB1* housekeeping gene for the determinaton of the *K. pneumoniae* relative amount in the feces, and the *bla*_CTX-M-15_ and *bla*_OXA-162_ genes for determining the relative amount of resistance genes compared to KP5825 were determined on the first and on the fifteenth days.

**Table 2:** Oligonucleotide probes and primers used in qPCR assays.

Oligonucleotides	Sequences
*rpoB1* forward primer	5′ CCC ACT ACG GTC GCG TAT G 3’
*rpoB1* reverse primer	5′ CAG ACC GAT GTT CGG ACC TT 3’
*rpoB1* probe	5′ VIC-CCG ATC GAA ACG CCT-MGB 3’
*oxa-162* forward primer	5′ GGG CGA ACC AAG CAT TTT T 3’
*oxa-162* reverse primer	5′ GCG ATC AAG CTA TTG GGA ATT T 3’
*oxa-162* probe	5′ FAM-CCC GCA TCT ACC TTT-MGB-NFQ 3’
*ctx-m-15* forward primer	5′ CGA CGT TAA ACA CCG CCA TT 3’
*ctx-m-15* reverse primer	5′ TGC CCG AGG TGA AGT GGT A 3’
*ctx-m-15* probe	5′ FAM-CGG GCG ATC CGC GTG-MGB-NFQ 3’

### Statistical analysis

Statistical analysis were performed using SSPS version 17.0 software (SPSS Inc., Chicago, IL, USA) and Microsoft Office Excel 2007 (Microsoft, Redmond, WA, USA). The variables such as the copy number of the *rpoB* housekeeping gene, *bla*_CTX-M-15_ and *bla*_OXA-161_ genes were compared by Wilcoxon rank-sum test. A *p*-value of less than 0.05 was considered statistically significant. P-values are represented by arterisks (*, p < 0.05; **, p < 0.001; ***, p < 0.0001).

### Ethics approval

Animals were maintained and handled in accordance with the recommendations of the Guidelines for the Care and Use of Laboratory Animals and the experiments were approved by the Animal Care Committee of Semmelweis University (Permission No. PE/EA/60-8/2018, PE/EA/964-5/2018).

### Consent to participate

Not applicable.

## Data Availability

The dataset supporting the conclusions of this article is included within the article.

## Abbreviations

BSL: Biosafety-level
CFU: Colony-forming unit
CPE: Carbapenemase-producing *Enterobacteriaceae*
CRKP: Carbapenem-resistant *Klebisella pneumoniae*
ESBL: Extended-spectrum β-lactamase
HGT: Horizontal gene transfer
HPLC: High-performance liquid chromatography
KP5825: *K. pneumoniae* Strain no. 5825
LoD: Limit of detection
LB: Luria -Bertani
MIC: Minimum inhibitory concentration
MDR: Multidrug resistant
NA: Not available
ST: Sequence type
